# Prevention of disordered eating in adolescents: the role of perfectionism and media internalisation

**DOI:** 10.1186/2050-2974-1-S1-O35

**Published:** 2013-11-14

**Authors:** Kelly Thompson, Kavitha Dorairaj, Simon Wilksch, Tracey Wade, Susan Paxton, S Bryn Austin, Sue Bryne

**Affiliations:** 1 School of Psychology, The University of Western Australia, Australia; 2 School of Psychology, Flinders University, Australia; 3 School of Psychological Science, LaTrobe University, Australia; 4 Department of Social and Behavioral Sciences, Harvard School of Public Health, USA; 5 Division of Adolescent and Young Adult Medicine, Boston Children's Hospital, USA

## 

There is extensive research into eating disorder risk factors, and recently the focus has moved to investigating the mechanisms underlying these factors. The current study examines the interrelationships between eating disorder symptoms and two proposed risk factors: perfectionism and media internalisation. This study uses data collected as part of the Prevention Across the Spectrum randomized controlled trial, which involves approximately 2000 Grade 7 and 8 adolescents across Australia. Students were randomly allocated to one of three eating disorder prevention programs or a control group. Students were assessed in 4 waves (pre-intervention, post-intervention, 6-month follow-up and 12-month follow-up) and the assessment included measures of perfectionism (Frost Multidimensional Perfectionism Scale), media internalisation (Sociocultural Attitudes Towards Appearance Questionnaire), and shape and weight concerns (Eating Disorder Examination Questionnaire). Preliminary analyses using a sample of baseline data suggest that the relationship between perfectionism and eating disorder symptoms is mediated by media internalisation, with differential effects depending upon the dimension of perfectionism and the outcome measure used in the analysis. Part two of this study will investigate the effects of the intervention programs on this relationship and outcome. The findings presented will have implications for our understanding of the development and prevention of eating disorder symptomatology.

This abstract was presented in the **Prevention** stream of the 2013 ANZAED Conference.

